# Supplementary material for: In Silico Analysis and Development of the Secretory Expression of D-Psicose-3-Epimerase in Escherichia coli
Source: Microorganisms. 2024 Aug 1;12(8):1574. doi: 10.3390/microorganisms12081574 (PMC11356227; doi:10.3390/microorganisms12081574)

**Figure S2.** The colony PCR result of the DPEase clones with: pET28a+DPEase (lane 1-4); pET28a+OmpA+DPEase (lane 5-8) and pET28a+PelB+DPEase (lane 9-12); M is DNA marker

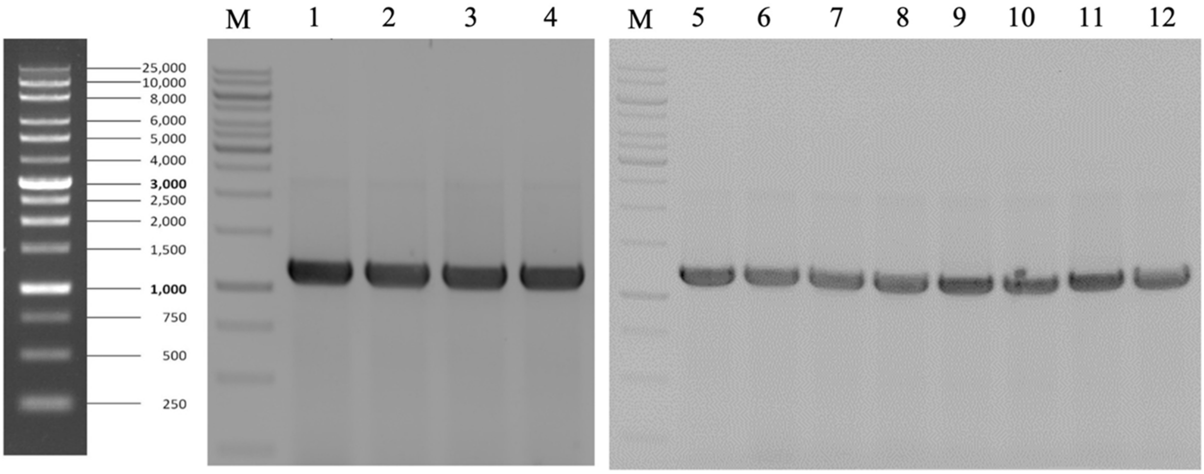

Supplement: Supplementary file 1 [file microorganisms-12-01574-s001.zip › Figure S2.pdf]
