# Supplementary material for: In Silico Analysis and Development of the Secretory Expression of D-Psicose-3-Epimerase in Escherichia coli
Source: Microorganisms. 2024 Aug 1;12(8):1574. doi: 10.3390/microorganisms12081574 (PMC11356227; doi:10.3390/microorganisms12081574)
Supplement: Supplementary file 1 [file microorganisms-12-01574-s001.zip › Supplementary data S1.pdf]

Supplementary Data S1. Amino acid sequences for DPEase without/with selected signal peptides used for localization and solubility predictions

> DPEase
MEHGIYYSYWEHEWSAKFGPYIEKVAKLGFDIIEVAAHHINEYSDAELATIRKSAKDNGIILTAGIGPSKTKNLSSEDA
AVRAAGKAFFERTLSNVAKLDIHTIGGALHSYWPIDYSQPVDKAGDYARGVEGINGIADFANDLGINLCIEVLNRFE
NHVLNTAAEGVAFVKDVGKNNVKVMLDTFHMNIEEDSFGDAIRTAGPLLGHFHTGESNRRVPGKGRMPWHEIGLA
LRDINYTGAVIMEPFVKTGGTIGSDIKVWRDLSGGADIAKMDEDARNALAFSRFVLGGCLEHHHHHH

>draA_DPEase
MKKLAIMAAASMVFAVSSAHAMEHGIYYSYWEHEWSAKFGPYIEKVAKLGFDIIEVAAHHINEYSDAELATIRKSA
KDNGIILTAGIGPSKTKNLSSEDAAVRAAGKAFFERTLSNVAKLDIHTIGGALHSYWPIDYSQPVDKAGDYARGVEGI
NGIADFANDLGINLCIEVLNRFENHVLNTAAEGVAFVKDVGKNNVKVMLDTFHMNIEEDSFGDAIRTAGPLLGHFH
TGESNRRVPGKGRMPWHEIGLALRDINYTGAVIMEPFVKTGGTIGSDIKVWRDLSGGADIAKMDEDARNALAFSRF
VLGGCLEHHHHHH

> faeG_DPEase
MKKTLIALAIAASAASGMAHAMEHGIYYSYWEHEWSAKFGPYIEKVAKLGFDIIEVAAHHINEYSDAELATIRKSAK
DNGIILTAGIGPSKTKNLSSEDAAVRAAGKAFFERTLSNVAKLDIHTIGGALHSYWPIDYSQPVDKAGDYARGVEGIN
GIADFANDLGINLCIEVLNRFENHVLNTAAEGVAFVKDVGKNNVKVMLDTFHMNIEEDSFGDAIRTAGPLLGHFHT
GESNRRVPGKGRMPWHEIGLALRDINYTGAVIMEPFVKTGGTIGSDIKVWRDLSGGADIAKMDEDARNALAFSRFV
LGGCLEHHHHHH

>fedA_DPEase
MKRLVFISFVALSMTAGSAMAMEHGIYYSYWEHEWSAKFGPYIEKVAKLGFDIIEVAAHHINEYSDAELATIRKSAK
DNGIILTAGIGPSKTKNLSSEDAAVRAAGKAFFERTLSNVAKLDIHTIGGALHSYWPIDYSQPVDKAGDYARGVEGIN
GIADFANDLGINLCIEVLNRFENHVLNTAAEGVAFVKDVGKNNVKVMLDTFHMNIEEDSFGDAIRTAGPLLGHFHT
GESNRRVPGKGRMPWHEIGLALRDINYTGAVIMEPFVKTGGTIGSDIKVWRDLSGGADIAKMDEDARNALAFSRFV
LGGCLEHHHHHH

> flgI_DPEase
MVIKFLSALILLLVTTAAQAMEHGIYYSYWEHEWSAKFGPYIEKVAKLGFDIIEVAAHHINEYSDAELATIRKSAKDN
GIILTAGIGPSKTKNLSSEDAAVRAAGKAFFERTLSNVAKLDIHTIGGALHSYWPIDYSQPVDKAGDYARGVEGINGI
ADFANDLGINLCIEVLNRFENHVLNTAAEGVAFVKDVGKNNVKVMLDTFHMNIEEDSFGDAIRTAGPLLGHFHTGE
SNRRVPGKGRMPWHEIGLALRDINYTGAVIMEPFVKTGGTIGSDIKVWRDLSGGADIAKMDEDARNALAFSRFVLG
GCLEHHHHHH

>malE_DPEase
MKIKTGARILALSALTTMMFSASALAMEHGIYYSYWEHEWSAKFGPYIEKVAKLGFDIIEVAAHHINEYSDAELATI
RKSAKDNGIILTAGIGPSKTKNLSSEDAAVRAAGKAFFERTLSNVAKLDIHTIGGALHSYWPIDYSQPVDKAGDYAR
GVEGINGIADFANDLGINLCIEVLNRFENHVLNTAAEGVAFVKDVGKNNVKVMLDTFHMNIEEDSFGDAIRTAGPLL
GHFHTGESNRRVPGKGRMPWHEIGLALRDINYTGAVIMEPFVKTGGTIGSDIKVWRDLSGGADIAKMDEDARNAL
AFSRFVLGGCLEHHHHHH

>OmpA_DPEase
MKKTAIAIAVALAGFATVAQAMEHGIYYSYWEHEWSAKFGPYIEKVAKLGFDIIEVAAHHINEYSDAELATIRKSAKD
NGIILTAGIGPSKTKNLSSEDAAVRAAGKAFFERTLSNVAKLDIHTIGGALHSYWPIDYSQPVDKAGDYARGVEGING
IADFANDLGINLCIEVLNRFENHVLNTAAEGVAFVKDVGKNNVKVMLDTFHMNIEEDSFGDAIRTAGPLLGHFHTG
ESNRRVPGKGRMPWHEIGLALRDINYTGAVIMEPFVKTGGTIGSDIKVWRDLSGGADIAKMDEDARNALAFSRFVL
GGCLEHHHHHH

> pbpG_DPEase
MPKFRVSLFSLALMLAVPFAPQAVAMEHGIYYSYWEHEWSAKFGPYIEKVAKLGFDIIEVAAHHINEYSDAELATIRK
SAKDNGIILTAGIGPSKTKNLSSEDAAVRAAGKAFFERTLSNVAKLDIHTIGGALHSYWPIDYSQPVDKAGDYARGVE
GINGIADFANDLGINLCIEVLNRFENHVLNTAAEGVAFVKDVGKNNVKVMLDTFHMNIEEDSFGDAIRTAGPLLGHF
HTGESNRRVPGKGRMPWHEIGLALRDINYTGAVIMEPFVKTGGTIGSDIKVWRDLSGGADIAKMDEDARNALAFSR
FVLGGCLEHHHHHH

> PelB_DPEase
MKYLLPTAAAGLLLLAAQPAMAMEHGIYYSYWEHEWSAKFGPYIEKVAKLGFDIIEVAAHHINEYSDAELATIRKS
AKDNGIILTAGIGPSKTKNLSSEDAAVRAAGKAFFERTLSNVAKLDIHTIGGALHSYWPIDYSQPVDKAGDYARGVE
GINGIADFANDLGINLCIEVLNRFENHVLNTAAEGVAFVKDVGKNNVKVMLDTFHMNIEEDSFGDAIRTAGPLLGHF
HTGESNRRVPGKGRMPWHEIGLALRDINYTGAVIMEPFVKTGGTIGSDIKVWRDLSGGADIAKMDEDARNALAFSR
FVLGGCLEHHHHHH

> xylF_DPEase
MKIKNILLTLCTSLLLTNVAAHAMEHGIYYSYWEHEWSAKFGPYIEKVAKLGFDIIEVAAHHINEYSDAELATIRKSA
KDNGIILTAGIGPSKTKNLSSEDAAVRAAGKAFFERTLSNVAKLDIHTIGGALHSYWPIDYSQPVDKAGDYARGVEGI
NGIADFANDLGINLCIEVLNRFENHVLNTAAEGVAFVKDVGKNNVKVMLDTFHMNIEEDSFGDAIRTAGPLLGHFH
TGESNRRVPGKGRMPWHEIGLALRDINYTGAVIMEPFVKTGGTIGSDIKVWRDLSGGADIAKMDEDARNALAFSRF
VLGGCLEHHHHHH

> yncJ_DPEase
MFTKALSVVLLTCALFSGQLMAMEHGIYYSYWEHEWSAKFGPYIEKVAKLGFDIIEVAAHHINEYSDAELATIRKSA
KDNGIILTAGIGPSKTKNLSSEDAAVRAAGKAFFERTLSNVAKLDIHTIGGALHSYWPIDYSQPVDKAGDYARGVEGI
NGIADFANDLGINLCIEVLNRFENHVLNTAAEGVAFVKDVGKNNVKVMLDTFHMNIEEDSFGDAIRTAGPLLGHFH
TGESNRRVPGKGRMPWHEIGLALRDINYTGAVIMEPFVKTGGTIGSDIKVWRDLSGGADIAKMDEDARNALAFSRF
VLGGCLEHHHHHH

> zraP_DPEase
MKRNTKIALVMMALSAMAMGSTSAFAMEHGIYYSYWEHEWSAKFGPYIEKVAKLGFDIIEVAAHHINEYSDAELA
TIRKSAKDNGIILTAGIGPSKTKNLSSEDAAVRAAGKAFFERTLSNVAKLDIHTIGGALHSYWPIDYSQPVDKAGDYA
RGVEGINGIADFANDLGINLCIEVLNRFENHVLNTAAEGVAFVKDVGKNNVKVMLDTFHMNIEEDSFGDAIRTAGPL
LGHFHTGESNRRVPGKGRMPWHEIGLALRDINYTGAVIMEPFVKTGGTIGSDIKVWRDLSGGADIAKMDEDARNA
LAFSRFVLGGCLEHHHHHH

> ampC_DPEase
MFKTTLCALLITASCSTFAMEHGIYYSYWEHEWSAKFGPYIEKVAKLGFDIIEVAAHHINEYSDAELATIRKSAKDNG
IILTAGIGPSKTKNLSSEDAAVRAAGKAFFERTLSNVAKLDIHTIGGALHSYWPIDYSQPVDKAGDYARGVEGINGIA
DFANDLGINLCIEVLNRFENHVLNTAAEGVAFVKDVGKNNVKVMLDTFHMNIEEDSFGDAIRTAGPLLGHFHTGES
NRRVPGKGRMPWHEIGLALRDINYTGAVIMEPFVKTGGTIGSDIKVWRDLSGGADIAKMDEDARNALAFSRFVLG
GCLEHHHHHH

> OmpC_DPEase
MKVKVLSLLVPALLVAGAANAMEHGIYYSYWEHEWSAKFGPYIEKVAKLGFDIIEVAAHHINEYSDAELATIRKSAK
DNGIILTAGIGPSKTKNLSSEDAAVRAAGKAFFERTLSNVAKLDIHTIGGALHSYWPIDYSQPVDKAGDYARGVEGIN
GIADFANDLGINLCIEVLNRFENHVLNTAAEGVAFVKDVGKNNVKVMLDTFHMNIEEDSFGDAIRTAGPLLGHFHT
GESNRRVPGKGRMPWHEIGLALRDINYTGAVIMEPFVKTGGTIGSDIKVWRDLSGGADIAKMDEDARNALAFSRFV
LGGCLEHHHHHH

> STII_DPEase
MKKNIAFLLASMFVFSIATNAYAMEHGIYYSYWEHEWSAKFGPYIEKVAKLGFDIIEVAAHHINEYSDAELATIRKSA
KDNGIILTAGIGPSKTKNLSSEDAAVRAAGKAFFERTLSNVAKLDIHTIGGALHSYWPIDYSQPVDKAGDYARGVEGI
NGIADFANDLGINLCIEVLNRFENHVLNTAAEGVAFVKDVGKNNVKVMLDTFHMNIEEDSFGDAIRTAGPLLGHFH
TGESNRRVPGKGRMPWHEIGLALRDINYTGAVIMEPFVKTGGTIGSDIKVWRDLSGGADIAKMDEDARNALAFSRF
VLGGCLEHHHHHH

> Ompf_DPEase
MMKRNILAVIVPALLVAGTANAMEHGIYYSYWEHEWSAKFGPYIEKVAKLGFDIIEVAAHHINEYSDAELATIRKSA
KDNGIILTAGIGPSKTKNLSSEDAAVRAAGKAFFERTLSNVAKLDIHTIGGALHSYWPIDYSQPVDKAGDYARGVEGI
NGIADFANDLGINLCIEVLNRFENHVLNTAAEGVAFVKDVGKNNVKVMLDTFHMNIEEDSFGDAIRTAGPLLGHFH
TGESNRRVPGKGRMPWHEIGLALRDINYTGAVIMEPFVKTGGTIGSDIKVWRDLSGGADIAKMDEDARNALAFSRF
VLGGCLEHHHHHH

> lamB_DPEase
MMITLRKLPLAVAVAAGVMSAQAMAMEHGIYYSYWEHEWSAKFGPYIEKVAKLGFDIIEVAAHHINEYSDAELATI
RKSAKDNGIILTAGIGPSKTKNLSSEDAAVRAAGKAFFERTLSNVAKLDIHTIGGALHSYWPIDYSQPVDKAGDYAR
GVEGINGIADFANDLGINLCIEVLNRFENHVLNTAAEGVAFVKDVGKNNVKVMLDTFHMNIEEDSFGDAIRTAGPLL
GHFHTGESNRRVPGKGRMPWHEIGLALRDINYTGAVIMEPFVKTGGTIGSDIKVWRDLSGGADIAKMDEDARNAL
AFSRFVLGGCLEHHHHHH

> araF_DPEase
MHKFTKALAAIGLAAVMSQSAMAMEHGIYYSYWEHEWSAKFGPYIEKVAKLGFDIIEVAAHHINEYSDAELATIRK
SAKDNGIILTAGIGPSKTKNLSSEDAAVRAAGKAFFERTLSNVAKLDIHTIGGALHSYWPIDYSQPVDKAGDYARGVE
GINGIADFANDLGINLCIEVLNRFENHVLNTAAEGVAFVKDVGKNNVKVMLDTFHMNIEEDSFGDAIRTAGPLLGHF
HTGESNRRVPGKGRMPWHEIGLALRDINYTGAVIMEPFVKTGGTIGSDIKVWRDLSGGADIAKMDEDARNALAFSR
FVLGGCLEHHHHHH

> nmpc_DPEase
MKKLTVAISAVAASVLMAMSAQAMEHGIYYSYWEHEWSAKFGPYIEKVAKLGFDIIEVAAHHINEYSDAELATIRKS
AKDNGIILTAGIGPSKTKNLSSEDAAVRAAGKAFFERTLSNVAKLDIHTIGGALHSYWPIDYSQPVDKAGDYARGVE

GINGIADFANDLGINLCIEVLNRFENHVLNTAAEGVAFVKDVGKNNVKVMLDTFHMNIEEDSFGDAIRTAGPLLGHF
HTGESNRRVPGKGRMPWHEIGLALRDINYTGAVIMEPFVKTGGTIGSDIKVWRDLSGGADIAKMDEDARNALAFSR
FVLGGCLEHHHHHH

> ppiA_DPEase
MFKSTLAAMAAVFALSALSPAAMAMEHGIYYSYWEHEWSAKFGPYIEKVAKLGFDIIEVAAHHINEYSDAELATIRK
SAKDNGIILTAGIGPSKTKNLSSEDAAVRAAGKAFFERTLSNVAKLDIHTIGGALHSYWPIDYSQPVDKAGDYARGVE
GINGIADFANDLGINLCIEVLNRFENHVLNTAAEGVAFVKDVGKNNVKVMLDTFHMNIEEDSFGDAIRTAGPLLGHF
HTGESNRRVPGKGRMPWHEIGLALRDINYTGAVIMEPFVKTGGTIGSDIKVWRDLSGGADIAKMDEDARNALAFSR
FVLGGCLEHHHHHH

> yaaI_DPEase
MKSVFTISASLAISLMLCCTAQAMEHGIYYSYWEHEWSAKFGPYIEKVAKLGFDIIEVAAHHINEYSDAELATIRKSA
KDNGIILTAGIGPSKTKNLSSEDAAVRAAGKAFFERTLSNVAKLDIHTIGGALHSYWPIDYSQPVDKAGDYARGVEGI
NGIADFANDLGINLCIEVLNRFENHVLNTAAEGVAFVKDVGKNNVKVMLDTFHMNIEEDSFGDAIRTAGPLLGHFH
TGESNRRVPGKGRMPWHEIGLALRDINYTGAVIMEPFVKTGGTIGSDIKVWRDLSGGADIAKMDEDARNALAFSRF
VLGGCLEHHHHHH

> glnH_DPEase
MKSVLKVSLAALTLAFAVSSHAMEHGIYYSYWEHEWSAKFGPYIEKVAKLGFDIIEVAAHHINEYSDAELATIRKSA
KDNGIILTAGIGPSKTKNLSSEDAAVRAAGKAFFERTLSNVAKLDIHTIGGALHSYWPIDYSQPVDKAGDYARGVEGI
NGIADFANDLGINLCIEVLNRFENHVLNTAAEGVAFVKDVGKNNVKVMLDTFHMNIEEDSFGDAIRTAGPLLGHFH
TGESNRRVPGKGRMPWHEIGLALRDINYTGAVIMEPFVKTGGTIGSDIKVWRDLSGGADIAKMDEDARNALAFSRF
VLGGCLEHHHHHH

> rna_DPEase
MKAFWRNAALLAVSLLPFSSANAMEHGIYYSYWEHEWSAKFGPYIEKVAKLGFDIIEVAAHHINEYSDAELATIRKS
AKDNGIILTAGIGPSKTKNLSSEDAAVRAAGKAFFERTLSNVAKLDIHTIGGALHSYWPIDYSQPVDKAGDYARGVE
GINGIADFANDLGINLCIEVLNRFENHVLNTAAEGVAFVKDVGKNNVKVMLDTFHMNIEEDSFGDAIRTAGPLLGHF
HTGESNRRVPGKGRMPWHEIGLALRDINYTGAVIMEPFVKTGGTIGSDIKVWRDLSGGADIAKMDEDARNALAFSR
FVLGGCLEHHHHHH

> DsbC_DPEase
MKKGFMLFTLLAAFSGFAQAMEHGIYYSYWEHEWSAKFGPYIEKVAKLGFDIIEVAAHHINEYSDAELATIRKSAK
DNGIILTAGIGPSKTKNLSSEDAAVRAAGKAFFERTLSNVAKLDIHTIGGALHSYWPIDYSQPVDKAGDYARGVEGIN
GIADFANDLGINLCIEVLNRFENHVLNTAAEGVAFVKDVGKNNVKVMLDTFHMNIEEDSFGDAIRTAGPLLGHFHT
GESNRRVPGKGRMPWHEIGLALRDINYTGAVIMEPFVKTGGTIGSDIKVWRDLSGGADIAKMDEDARNALAFSRFV
LGGCLEHHHHHH

> rbsB_DPEase
MNMKKLATLVSAVALSATVSANAMAMEHGIYYSYWEHEWSAKFGPYIEKVAKLGFDIIEVAAHHINEYSDAELATI
RKSAKDNGIILTAGIGPSKTKNLSSEDAAVRAAGKAFFERTLSNVAKLDIHTIGGALHSYWPIDYSQPVDKAGDYAR
GVEGINGIADFANDLGINLCIEVLNRFENHVLNTAAEGVAFVKDVGKNNVKVMLDTFHMNIEEDSFGDAIRTAGPLL
GHFHTGESNRRVPGKGRMPWHEIGLALRDINYTGAVIMEPFVKTGGTIGSDIKVWRDLSGGADIAKMDEDARNAL
AFSRFVLGGCLEHHHHHH

> gfcA_DPEase
MKHKLSAILMAFMLTTPAAFAMEHGIYYSYWEHEWSAKFGPYIEKVAKLGFDIIEVAAHHINEYSDAELATIRKSAK
DNGIILTAGIGPSKTKNLSSEDAAVRAAGKAFFERTLSNVAKLDIHTIGGALHSYWPIDYSQPVDKAGDYARGVEGIN
GIADFANDLGINLCIEVLNRFENHVLNTAAEGVAFVKDVGKNNVKVMLDTFHMNIEEDSFGDAIRTAGPLLGHFHT
GESNRRVPGKGRMPWHEIGLALRDINYTGAVIMEPFVKTGGTIGSDIKVWRDLSGGADIAKMDEDARNALAFSRFV
LGGCLEHHHHHH
