# Supplementary material for: In Silico Analysis and Development of the Secretory Expression of D-Psicose-3-Epimerase in Escherichia coli
Source: Microorganisms. 2024 Aug 1;12(8):1574. doi: 10.3390/microorganisms12081574 (PMC11356227; doi:10.3390/microorganisms12081574)
Supplement: Supplementary file 1 [file microorganisms-12-01574-s001.zip › Supplementary data S2.pdf]

Supplementary Data S2

The condon optimized D-psicose-3-epimerase (DPEase) gene for this study

```
Optimized   1  CACGGTATTTACTATAGCTACTGGGAGCACGAATGGAGCGCGAAATTCGGCCCGTATATC
Original    1  CACGGCATCTATTATTCTTACTGGGAACATGAGTGGAGCGCCAAGTTCGGTCCCTATATC

Optimized  61  GAGAAGGTTGCGAAACTGGGTTTTGACATCATTGAAGTGGCGGCGCACCACATTAACGAG
Original   61  GAGAAGGTCGCCAAGCTCGGTTTCGACATCATCGAAGTCGCCGCCCACCATATCAACGAA

Optimized 121  TACAGCGATGCGGAACTGGCGACCATCCGTAAGAGCGCGAAAGACAACGGCATCATTCTG
Original  121  TACAGCGACGCCGAACTCGCGACCATCAGGAAGAGCGCGAAGGATAACGGCATCATCCTC

Optimized 181
ACCGCGGGTATTGGCCCGAGCAAGACCAAAAACCTGAGCAGCGAGGACGCGGCGGTTCGT
Original  181  ACCGCCGGCATCGGTCCGTCGAAAACCAAGAACCTGTCGTCGGAAGATGCTGCGGTGCGT

Optimized 241
GCGGCGGGTAAAGCGTTCTTTGAACGTACCCTGAGCAACGTGGCGAAGCTGGACATCCAC
Original  241  GCGGCCGGCAAGGCGTTCTTTGAAAGAACCCTTTCGAACGTCGCCAAGCTCGATATCCAC

Optimized 301
ACCATTGGTGGCGCGCTGCACAGCTACTGGCCGATCGACTATAGCCAGCCGGTTGACAAG
Original  301  ACCATCGGCGGCGCATTGCATTCCTATTGGCCAATCGATTATTCGCAGCCCGTCGACAAG

Optimized 361
GCGGGCGATTATGCGCGTGGTGTGGAGGGCATCAACGGTATTGCGGACTTCGCGAACGAT
Original  361  GCAGGCGATTATGCGCGCGGCGTCGAGGGTATCAACGGCATTGCCGATTTCGCCAATGAT

Optimized 421  CTGGGTATCAACCTGTGCATTGAGGTTCTGAACCGTTTCGAGAACCATGTGCTGAACACC
Original  421  CTCGGCATCAACCTGTGCATCGAAGTCCTCAACCGCTTTGAAAACCACGTCCTCAACACG

Optimized 481  GCGGCGGAAGGCGTGGCGTTTGTTAAGGATGTGGGTAAAAACAACGTGAAGGTTATGCTG
Original  481  GCGGCGGAAGGCGTCGCTTTTGTGAAGGATGTCGGCAAGAACAATGTGAAAGTCATGCTG

Optimized 541  GACACCTTCCACATGAACATTGAAGAGGACAGCTTTGGTGATGCGATCCGTACCGCGGGT
Original  541  GATACCTTCCACATGAACATCGAGGAAGACAGTTTCGGTGACGCCATCCGCACGGCCGGC

Optimized 601
CCGCTGCTGGGTCACTTCCACACCGGCGAGAGCAACCGTCGTGTTCCGGGTAAAGGCCGT
Original  601  CCGCTTCTGGGGCACTTCCATACCGGTGAAAGCAATCGCCGCGTACCGGGCAAGGGCAGA

Optimized 661  ATGCCGTGGCATGAAATTGGCCTGGCGCTGCGTGATATTAACTACACCGGTGCGGTTATC
Original  661  ATGCCGTGGCACGAAATCGGCCTTGCGCTGCGTGATATCAACTACACCGGCGCGGTAATC

Optimized 721  ATGGAGCCGTTTGTGAAAACCGGTGGCACCATCGGTAGCGACATTAAGGTTTGGCGTGAT
Original  721  ATGGAGCCTTTCGTCAAGACAGGCGGCACCATCGGCTCGGATATCAAGGTGTGGCGCGAC

Optimized 781
CTGAGCGGTGGCGCGGACATCGCGAAGATGGACGAAGATGCGCGTAACGCGCTGGCGTTC
Original  781  CTGAGCGGTGGCGCCGACATCGCGAAAATGGATGAAGATGCCCGCAATGCGCTGGCATTC

Optimized 841  AGCCGTTTTGTGCTGGGTGGCTGC
Original  841  TCCCGCTTCGTTCTTGGTGGCTGT
```
