# Supplementary figures and images for: In Silico Analysis and Development of the Secretory Expression of D-Psicose-3-Epimerase in Escherichia coli
Source: Microorganisms. 2024 Aug 1;12(8):1574. doi: 10.3390/microorganisms12081574 (PMC11356227; doi:10.3390/microorganisms12081574)

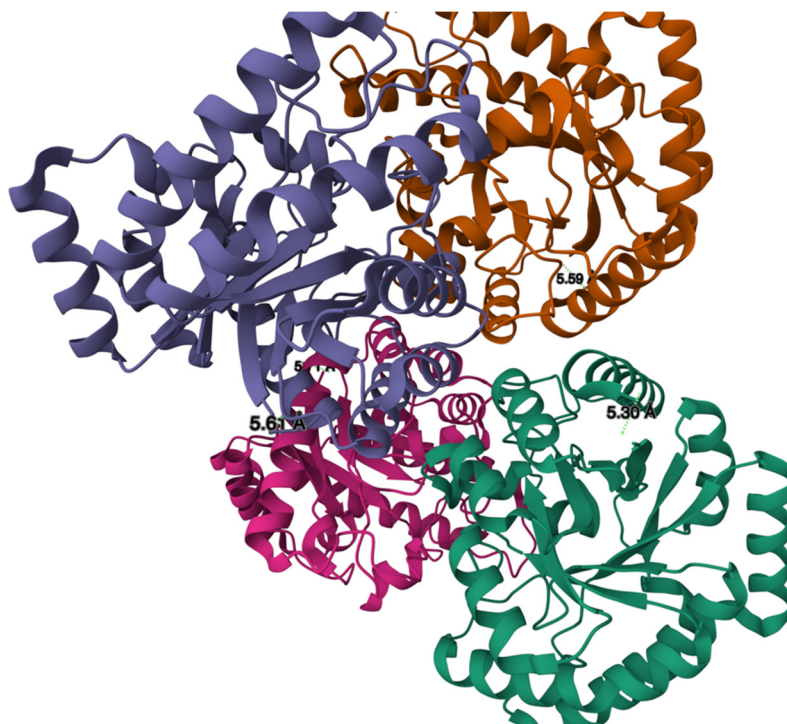

Figure S1. N and C terminal distance measurement on DPEase structure (Uniprot identifier: A9CH28).

Supplement: Supplementary file 1 [file microorganisms-12-01574-s001.zip › Figure S1.pdf]
